# RETRACTION: Effect of Endoscopic Loop Ties in Acute Appendicitis on Wound Infection Tate: A Meta‐Analysis

**DOI:** 10.1111/iwj.70215

**Published:** 2025-02-11

## Abstract

**RETRACTION**: DingH. and LiY. , “Effect of Endoscopic Loop Ties in Acute Appendicitis on Wound Infection Tate: A Meta‐Analysis,” International Wound Journal 20, no. 8 (2023): 3048–3056, 10.1111/iwj.14180.37165758 PMC10502295

The above article, published online on 10 May 2023, in Wiley Online Library (http://onlinelibrary.wiley.com/), has been retracted by agreement between the journal Editor in Chief, Professor Keith Harding; and John Wiley & Sons Ltd. Following an investigation by the publisher, all parties have concluded that this article was accepted solely on the basis of a compromised peer review process. The editors have therefore decided to retract the article. The authors did not respond to our notice regarding the retraction.

**RETRACTION**: H. Ding and Y. Li , “Effect of Endoscopic Loop Ties in Acute Appendicitis on Wound Infection Tate: A Meta‐Analysis,” International Wound Journal 20, no. 8 (2023): 3048–3056, 10.1111/iwj.14180.37165758 PMC10502295

The above article, published online on 10 May 2023, in Wiley Online Library (http://onlinelibrary.wiley.com/), has been retracted by agreement between the journal Editor in Chief, Professor Keith Harding; and John Wiley & Sons Ltd. Following an investigation by the publisher, all parties have concluded that this article was accepted solely on the basis of a compromised peer review process. The editors have therefore decided to retract the article. The authors did not respond to our notice regarding the retraction.

